# Sarcopenia and Nutrition in Elderly Rheumatoid Arthritis Patients: A Cross-Sectional Study to Determine Prevalence and Risk Factors

**DOI:** 10.3390/nu15112440

**Published:** 2023-05-24

**Authors:** Laura Cano-García, Sara Manrique-Arija, Carmen Domínguez-Quesada, Juan Crisóstomo Vacas-Pérez, Pedro J. Armenteros-Ortiz, Desiré Ruiz-Vilchez, José María Martín-Martín, Rocío Redondo-Rodríguez, Aimara García-Studer, Fernando Ortiz-Márquez, Natalia Mena-Vázquez, Antonio Fernández-Nebro

**Affiliations:** 1 Instituto de Investigación Biomédica de Málaga (IBIMA)-Plataforma Bionand, 29010 Málaga, Spain; lauracano.due@gmail.com (L.C.-G.); sarama_82@hotmail.com (S.M.-A.); rocioredondo91@hotmail.com (R.R.-R.); aimara.garcia95@gmail.com (A.G.-S.); fomquez@gmail.com (F.O.-M.); afnebro@gmail.com (A.F.-N.); 2 UGC de Reumatología, Hospital Regional Universitario de Málaga, 29009 Málaga, Spain; 3 Departamento de Medicina, Universidad de Málaga, 29010 Málaga, Spain; 4 UGC de Reumatología, Hospital Universitario Virgen Macarena, 41701 Sevilla, Spain; cdq.due@gmail.com; 5 UGC de Reumatología, Hospital Universitario Reina Sofía de Córdoba, 14004 Córdoba, Spain; juancris61@hotmail.com (J.C.V.-P.); pedroj.armemteros.sspa@juntadeandalucia.es (P.J.A.-O.); desiree.ruiz@hotmail.com (D.R.-V.); 6 Instituto Maimónides de Investigación Biomédica de Córdoba (IMIBIC), 14004 Córdoba, Spain; 7 UGC de Reumatología, Hospital Universitario Nuestra Señora de la Candelaria, 38010 Santa Cruz de Tenerife, Spain; chema904@hotmail.com

**Keywords:** rheumatoid arthritis, elderly, obesity, sarcopenia, nutrition, malnutrition, body composition

## Abstract

Objective: To describe the prevalence of sarcopenia in rheumatoid arthritis (RA) patients aged ≥65 years and identify the risk factors associated with sarcopenia. Methods: This is a multicenter, controlled, cross-sectional study of 76 RA patients and 76 age- and sex-matched healthy controls. Sarcopenia was defined according to the revised criteria of the European Working Group on Sarcopenia in Older People (EWGSOP2). Whole-body dual-energy X-ray absorptiometry (DXA) was performed. Binary regression was used to assess the relationship between sarcopenia and sex, age, duration of RA, Mini Nutritional Assessment (MNA) score, and Short Physical Performance Battery (SPPB) score in patients with RA. Results: Nearly 80% of participants were female, and the average age was >70 years. Patients with RA had lower muscle mass and greater adiposity (fat-to-muscle ratio mean [SD] 0.9 [0.2] vs. 0.8 [0.2]; *p* = 0.017) than controls, mainly in the central area (android/gynoid ratio, median [p25–p75]: 1.0 [0.9–1.2] vs. 0.9 [0.8–1.1]; *p* < 0.001). Twelve patients (15.8%) and three controls (3.9%) had confirmed sarcopenia (*p* = 0.014). Sarcopenic obesity was observed in 8/76 patients with RA (10.5%) and in 1/76 controls (1.3%) (*p* = 0.016). The factors associated with sarcopenia were male sex (OR [95% CI]: 9.3 [1.1–80.4]; *p* = 0.042), disease duration (OR [95% CI]: 1.1 [1.0–1.2]; *p* = 0.012), and nutritional status according to the MNA (OR [95% CI]: 0.7 [0.5–0.9]; *p* = 0.042). Conclusions: Our results suggest that patients with RA aged ≥65 years may be at increased risk for sarcopenia, adiposity, and malnutrition (especially male patients with long-standing disease) and have poor nutritional status.

## 1. Introduction

Rheumatoid arthritis (RA) is an immune-mediated disease characterized by chronic synovitis, joint destruction, disability, and decreased life expectancy. RA can occur at any time in a person’s life, although its frequency increases with age. Inflammation is more poorly controlled, even with treatment, in patients whose disease first appears after age 60 years than in younger individuals [1]. In addition, older patients tend to accumulate more comorbidities and have worse arthritis-related health outcomes [2,3]. In the last 20 years, the availability of biological therapies has enabled patients with RA to gain life years [4]. However, longer survival does not necessarily indicate good health, as years gained do not necessarily imply ability. This is particularly relevant for individuals aged ≥65 years because they tend to accumulate other conditions that lead to a progressive loss of autonomy and contribute to the development of geriatric syndromes, such as sarcopenia, malnutrition, frailty, and cognitive impairment. These conditions can generate a significant social and economic burden [5,6].

After 60 years of age, skeletal muscle mass decreases by 3% each year, therefore an 80-year-old person would have up to 50% of the muscle mass they had at 40 years of age [7]. Sarcopenia is characterized by a loss of skeletal muscle mass and strength. It is generally associated with aging, chronic disease and predicts disability, and an increased incidence of falls, fractures, hospitalization, and death [8,9,10]. Although sarcopenia is a multifactorial phenomenon associated with age, it may be more prevalent in patients with RA or other systemic diseases [5,11]. Previous studies have shown that 20–40% of RA patients have sarcopenia [5,11,12,13]. However, very few studies use the new, revised criteria of “The European Working Group on Sarcopenia in Older People (EWGSOP2)”, which are considered to be more accurate and specific for the diagnosis of sarcopenia [14], and have been widely adopted by researchers and health care professionals. In addition, no studies have focused on elderly patients with RA using the new criteria. The factors associated with sarcopenia in RA, include advanced age [15], inflammatory activity [11,16], glucocorticoids [17], sedentary lifestyle, and low intake of disease-modifying drugs (DMARDs) [15]. Furthermore, chronic pain and increased energy expenditure during rest can also contribute to decreased muscle mass in RA. The prevalence of sarcopenia in RA patients aged over 65 years is almost three times greater than in younger individuals [18,19,20,21].

Although sarcopenia entails a reduction in muscle mass, it is not always associated with a decrease in body mass index (BMI), because over time there is an increase in adiposity, especially around the trunk and viscera. This situation can lead to what is known as sarcopenic obesity, which has been estimated to affect 12.6% of patients with RA [22]. Sarcopenic obesity leads to other metabolic changes and is linked to aging, lack of exercise, and the protein, hypercatabolism, which results from elevated levels of proinflammatory cytokines and a sedentary lifestyle [23].

Sarcopenia can also coexist with protein-calorie malnutrition due to nutritional imbalances associated with poor dental health, loss of taste and appetite, adverse drug effects, and social isolation [24]. It has been reported that up to two thirds of patients with RA have inadequate dietary intake and may develop malnutrition, yet very few studies address nutrition in patients with RA [25].

It is therefore important to understand the epidemiology of comorbidities, polypharmacy, and geriatric syndromes in older RA patients in order to provide more effective care and maintain functional independence without deviating too far from strategies that ensure strict control of the disease. However, data on the factors associated with sarcopenia in the context of adiposity and malnutrition are limited, specifically among older RA patients. Therefore, the objectives of our study were as follows: (1) to describe the prevalence of sarcopenia and sarcopenic obesity in RA patients aged ≥65 years; and (2) to identify the risk factors associated with sarcopenia in these patients.

## 2. Materials and Methods

### 2.1. Study Design, Data Source, and Sample

This is a multicenter, controlled, cross-sectional study. Data were obtained from four Spanish rheumatology university hospitals (Hospital Regional Universitario de Málaga, Hospital Universitario Virgen Macarena, Hospital Universitario Reina Sofía, and Hospital Universitario Virgen de Candelaria) through specialist consultations held by rheumatologists and nurses specialized in the management of RA. All participants provided their written informed consent before entering the study. The study was conducted according to the principles of the Declaration of Helsinki, and the protocol was approved by the Ethics Committee of Málaga (code: 2406-N-20).

#### 2.1.1. Patients

All participating patients were recruited consecutively between April and December 2021. Patients with RA were selected according to the 2010 criteria of ACR/EULAR [26] and were aged ≥65 years at inclusion. Onset of disease had to be after the age of 16 years. Patients with inflammatory diseases other than RA (except secondary Sjögren syndrome) or active infection were excluded.

#### 2.1.2. Controls

The voluntary controls were unrelated persons of the same age (±3 years), and sex, as the patient belonging to the patient’s social or family environment. Controls must not have had any systemic inflammatory or autoimmune diseases or symptoms indicative of such a disease.

### 2.2. Study Protocol

RA patients from participating centers are usually followed up and treated jointly by a rheumatologist and a nurse at specific consultations every 3–6 months, or more frequently if clinically necessary. The reference rheumatologist invited the patients to participate in the study and, after obtaining their written informed consent, confirmed the selection criteria and collected the clinical data following a collection protocol that was specifically designed for this study. In the nursing office, questionnaires were completed and anthropometric measurements were taken. Biological samples were extracted after at least 8 h of fasting, and a whole-body dual-energy X-ray absorptiometry (DXA) scan was performed.

#### Body Composition by DXA

Body composition was measured using DXA equipment from two different manufacturers. Two of the centers used the GE Lunar Prodigy DXA device and the other two used the Hologic Discovery device. Each device was calibrated according to the manufacturer’s specifications using a lumbar spine phantom and operated in the highest resolution mode. Participants were scanned in the supine position, as indicated by the manufacturer. The body composition measurements for the participants generated by the Hologic Discovery device were adjusted based on the method recommended by Shepherd et al. [27]. The coefficient of variation was less than 1%.

### 2.3. Variables and Definitions

#### 2.3.1. Sarcopenia, Sarcopenic Obesity, and Osteosarcopenia

The primary endpoint of the study was sarcopenia, as defined in the revised 2019 criteria of EWGSOP2 [14]. Outcomes were classified as follows: (1) probable sarcopenia, i.e., only low muscle strength (<27 kg in men and <16 kg in women); (2) confirmed sarcopenia, i.e., low muscle strength and low muscle quantity (appendicular lean mass index [ALM/height^2^] < 7.0 kg/m^2^ in men and <5.5 kg/m^2^ in women); and (3) severe sarcopenia, i.e., low muscle strength, low muscle quantity/quality, and poor physical performance.

Sarcopenic obesity was defined as the simultaneous presence of sarcopenia and obesity using the sex-specific cut-off values for the body fat mass index (FMI) developed by Kelly et al. [28] with FMI ≥ 13 for women and ≥9 for men.

Osteoporosis in the hip and spine was defined according to the 1994 World Health Organization (WHO) criteria [29]. Additionally, osteosarcopenia was defined as fulfilling both definitions (sarcopenia and osteoporosis in the hip or spine) simultaneously.

#### 2.3.2. Demographics

Age, sex, educational level, income level, toxic habits, comorbidities, and drug use were collected from all participants. The comorbidities recorded were those related to traditional cardiovascular risk factors (smoking, obesity, arterial hypertension, diabetes mellitus, dyslipidemia, history of cardiovascular disease, and sedentary lifestyle), those included in the Charlson Comorbidity Index (CCI) [30], and others, such as osteoporosis. The crude and age-adjusted CCI (age-CCI) were calculated [31]. An age-CCI >2 has been associated with a lower 10-year survival rate, which decreases proportionally with an increasing age-CCI value (i.e., 1, 2, 3, 4, 5, 6, and ≥7 are associated with survival rates of 96%, 90%, 77%, 53%, 21%, 2%, and 0%, respectively) [30]. Data were specifically collected on analgesics, non-steroidal anti-inflammatory drugs (NSAIDs), glucocorticoids, conventional synthetic DMARDs (csDMARDs), and biological DMARDs (bDMARDs). Polypharmacy was defined as the routine use of ≥5 medications, according to the WHO 2019 criteria [32].

#### 2.3.3. Anthropometric Variables, Body Composition, Strength, Performance, and Nutrition

Weight (kg), height (m), arm circumference (cm), and triceps skinfold (mm) were recorded for all participants. Underweight, overweight, and obesity were classified according to the BMI categories of the WHO [33].

The body composition data collected included total and regional (trunk and appendicular) masses (kg), fat mass (FM) (kg), lean mass (LM) (kg), and bone mass content (BMC) (g). Adiposity was defined as the percentage of total body fat. The FMI and fat-free mass index (FFMI) were calculated as FM and fat-free mass (kg) divided by height (m^2^), respectively. Additionally, the percentage of fat mass in android and gynoid tissues was measured, and fat distribution was determined by calculating the ratio of fat in the android region to fat in the gynoid region (A/G ratio). To calculate ALM, the lean mass of the four limbs was added, assuming that all non-fat and non-bone tissue is skeletal muscle ALM [34]. The fat-to-muscle ratio (FMR) was calculated as the total FM (kg) divided by the total LM (kg) obtained by DXA, with adjustment for bone mineral content [35].

The presence of sarcopenia was investigated using the five-item SARC-F (Strength, Assistance in walking, Rise from a chair, Climb stairs, and Falls) questionnaire, which assesses participants’ strength, functional capacity, and frailty [36]. Each item is scored on a scale of 0 to 2, resulting in a total score ranging from 0 to 10 points. Frailty, functional capacity, and strength were also evaluated using the Short Physical Performance Battery (SPPB) [37], which evaluates lower extremity function in older adults through three domains (walking, sit-to-stand, and balance), with a score ranging from 0 to 12 points. A higher score on the SPPB suggests superior physical function. A score of less than 10 points on the SPPB is indicative of a decline in physical performance, as per the Spanish National Health System’s guidelines [38] and EWGSOP2 (16). An SPPB ≥3 and ≤9 was considered physical frailty, but not mobility impairment [39]. Furthermore, handgrip strength was measured on the dominant hand using a dynamometer (CAMRY™ EH101) with a maximum weight capacity of 90 kg.

Physical activity was assessed using the International Physical Activity Questionnaire (IPAQ) [40], the Health Assessment Questionnaire (HAQ) [41], and the Steinbrocker classification [42]. Quality of life was evaluated using the EuroQoL 5-D (EQ-5D) health-related quality of life questionnaire [43].

Malnutrition and risk of malnutrition were evaluated using the Short-form Mini Nutritional Assessment (MNA-SF) screening tool (range, 0 to 14), which scores as follows: normal, >11; risk of malnutrition, 8–11; and malnutrition, 0–7 points [44]. Additionally, several analytical parameters were measured, including complete blood count, vitamins D and B12, total protein, albumin, and C-reactive protein.

#### 2.3.4. RA Variables

Inflammatory activity in RA patients was assessed using the 28-joint Disease Activity Score with erythrocyte sedimentation rate (DAS28-ESR) (continuous, range 0–9.4) [45]. Activity according to the DAS28-ESR was classified as follows: high, >5.1; moderate, 3.2–5.1; low, 2.6–3.2; and remission ≤ 2.6. Severity of RA was assessed based on the presence of rheumatoid factor (RF) (positive, >10 IU/mL), anti–citrullinated peptide antibody (ACPA) (positive, >20 IU/mL), and the presence of radiological erosions, defined as ≥1 radiographic erosion in the joints of the hands and feet.

### 2.4. Statistical Analysis

A comprehensive descriptive analysis of the main variables was performed. The frequencies of the qualitative variables were reported as the number of observations and their corresponding percentages. Quantitative variables were presented as mean ± standard deviation or as median (25th and 75th percentiles) if they were not normally distributed (Kolmogorov–Smirnov test). Characteristics were compared between RA patients and controls using the appropriate statistical test, such as the Pearson χ^2^ test (or Fisher’s exact test when applicable) or the t test. Furthermore, a bivariate analysis was carried out to examine the relationship between sarcopenia and other variables, in both the overall sample and RA subgroup. A binomial logistic regression model was developed to identify factors associated with the risk of sarcopenia (dependent variable). The variables that were significant in the bivariate analysis and those of clinical relevance were included as factors in the model (age, male sex, duration of RA, DAS28-ESR, MNA, and SPPB) using backward selection. The multicollinearity of the independent variables was examined using the Pearson correlation coefficient, and variables with an r-coefficient greater than 0.4 were included in the model separately, with the best-performing variable selected to explain the dependent variable. All variables that reached a *p*-value of less than 0.51 were included in the multivariate models, and a *p*-value less than 0.05 was considered statistically significant. The statistical analyses were performed using IBM SPSS Statistics for Mac OS, Version 28.0 (IBM Corp., Armonk, NY, USA).

## 3. Results

### 3.1. Characterization of RA and Controls

#### 3.1.1. Demographics

The study sample consisted of 76 patients with RA who were aged ≥65 years, and 76 age and sex-matched controls without RA. As displayed in Table 1, the two groups were well-balanced in terms of age and sex. Nearly 80% of participants were female, and the average age was over 70 years with most participants falling within the 70- to 80-year age group. Patients had lower levels of formal education and income than controls. Although there were no significant differences in smoking habits, there was a higher proportion of ex-smokers among RA patients. Additionally, RA patients had a higher prevalence of comorbidities, such as hypertension, osteoporosis, and asthma, as well as higher CCI values, irrespective of whether these were crude or age adjusted. Based on the results, 48/76 RA patients (63.1%) and 40/76 controls (52.6%) would have an age-adjusted 10-year survival of 2% or less (*p* = 0.003). Numerically, there were more RA patients with dyslipidemia, although the difference was not statistically significant.

#### 3.1.2. Characteristics Associated with RA

Patients had a long history of disease and a high proportion of positive antibody levels (Table 2). Serum CRP levels remained within the reference range in both groups, although they were higher in patients with RA. While most patients were in remission or had low disease activity at the time of the study, 27/76 (35.5%) continued to have moderate or high disease activity (as measured using the DAS28-ESR) and HAQ values indicating moderate disability (mean, 1.282). This clinical situation was observed even though 59.2% of patients (45/76) were taking csDMARDs and 73.7% (56/76) were taking bDMARDs after failure of ≥1 DMARD. Moreover, many more patients than controls were polymedicated (90.8% vs. 30.3%; *p* < 0.001) and were receiving more drugs (median [p25–p75], 8.0 [6.0–11.0] vs. 3.0 [2.0–4.7]; *p* < 0.001). All participants consumed NSAIDs and analgesics, although this was more common among the patients.

#### 3.1.3. Anthropometric Measurements, Nutritional Status, Strength, and Physical Performance in Patients with RA and Controls

As shown in Table 3, no anthropometric or nutritional differences were recorded between cases and controls, except that patients with RA had slightly lower triceps skinfold thickness and lower vitamin B12 levels than controls. However, all values for strength, performance, and health-related quality of life were clearly worse in the cases than in the controls. RA patients experienced greater functional limitation than controls, although most were able to carry out their activities of daily living by themselves (Steinbrocker class II, 50.0%). The results of the IPAQ showed that RA patients did less than half as much physical exercise as controls (median [p25–p75] METs, 260.0 [0.0–630.0] vs. 594.0 [0.0–1173.7]; *p* = 0.002). In addition, RA patients had poorer physical performance according to the SPPB, and 48/76 patients (84.2%) were physically frail, compared with only 18/76 controls (34.6%) (*p* < 0.001).

#### 3.1.4. Analysis of Body Composition and Prevalence of Sarcopenia in RA Patients and Controls

As shown in Table 3, the prevalence of confirmed sarcopenia based on the EWGSOP2 criteria was higher in RA patients than in controls: 12/76 (15.8%) vs. 3/76 (3.9%) (*p* = 0.014). While many more RA patients than controls (46.1% vs. 14.5%) had probable sarcopenia (*p* < 0.001), severe sarcopenia was recorded in only one patient and no controls. Sarcopenic obesity was present in 8/76 RA patients (10.5%) and 1/76 controls (1.3%) (*p* = 0.016). Osteosarcopenia was recorded in only 3/76 RA patients (4.1%) and in no controls (*p* = 0.076).

Supplementary Table S1 shows the results for body composition in the two subgroups (total body and individual areas). Adiposity tended to be more frequent in RA patients than in controls (*p* = 0.097), and fat content in terms of fat mass (FMR) was greater (mean [SD]: 0.9 [0.2] vs. 0.8 [0.2]; *p* = 0.017) and BMC lower (*p* = 0.006). Moreover, compared with controls, RA patients had a lower appendicular FFMI (g/m^2^) (median [p25–p75]: 6.0 [5.3–7.0] vs. 6.2 [5.6–7.2]; *p* = 0.074), a lower gynoid mass percentage (*p* = 0.005), and greater abdominal mass (A/G ratio, median [p25–p75]: 1.0 [0.9–1.2] vs. 0.9 [0.8–1.1]; *p* < 0.001).

### 3.2. Characteristics of RA Patients with Sarcopenia

Supplementary Table S2 shows that, compared with non-sarcopenic RA patients, sarcopenic RA patients were somewhat older (*p* = 0.095) and were less frequently in remission or had low disease activity (*p* = 0.072) approaching significance. RA patients had a longer duration of RA (*p* = 0.018). As for nutritional status, poorer results in MNA screening were recorded for sarcopenic RA patients (*p* = 0.029) who were also more frequently malnourished (*p* = 0.030). In terms of physical functioning, there were few differences between the groups, except that sarcopenic RA patients did less exercise according to the IPAQ (*p* = 0.002) and had a lower SPPB (*p* = 0.040). Patients, with sarcopenic obesity compared with non-sarcopenic obesity RA patients, were also more frequently malnourished (*p* = 0.047) and had a reduced handgrip strength (*p* = 0.001) (Supplementary Table S3).

Supplementary Table S4 shows the results for body composition (total and by area) in sarcopenic and non-sarcopenic patients with RA. Sarcopenic RA patients had a lower total body mass than non-sarcopenic RA patients (TM, median [p25–p75]: 65.3 kg [52.5–74.5] vs. 73.4 kg [65.3–83.7]; *p* = 0.019); this was generally seen as lower values for FM, BMC, and, above all, FFM for all the areas, although especially in the limbs (appendicular LM, median [p25–p75]: 12.6 kg [11.2–13.9] vs. 14.8 kg [13.1–18]; *p* < 0.001). Supplementary Table S5 shows the results for body composition in sarcopenic obesity and non-sarcopenic obesity in RA. Sarcopenic obesity patients had a lower appendicular LM and FFMI than non-sarcopenic RA patients.

### 3.3. Factors Associated with Sarcopenia in RA

Table 4 presents the results of the univariate and multivariate analyses of the clinical-epidemiological variables associated with sarcopenia, taking into account only the RA patients. In the univariate analysis, the risk of confirmed sarcopenia in RA patients varied significantly with age, sex, duration of RA, degree of disease activity according to the DAS28-ESR, and the results of the MNA screening test and SPPB.

**Figure 1:**
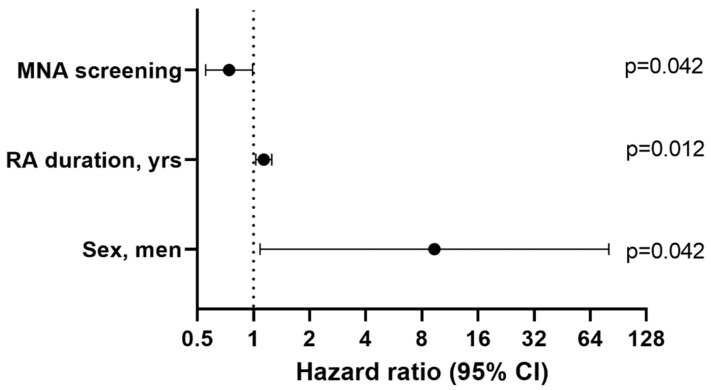
Logistic regression analysis plot. Dependent variable: confirmed sarcopenia in patients with RA. Abbreviation: MNA = Mini Nutritional Assessment.

The results of the multivariate analysis (Figure 1) showed that the risk of sarcopenia in RA patients was 9.3 times higher in men than in women, and that this increased by 10% per year with RA and fell by 30% for each point of nutritional improvement in the MNA. Sex, RA duration, and the risk of malnutrition evaluated by the MNA screening tool explain 72.2% of the total cumulative variance in sarcopenia. Supplementary Table S6 showed the results of the univariate and multivariate that the risk for sarcopenic obesity in RA patients (Supplementary Figure S1). Age and RA duration explain 73.6% of the total cumulative variance in sarcopenic obesity.

## 4. Discussion

Our results suggest that RA patients aged ≥65 years may require complex disease management and integrated care covering both comorbid conditions and RA. The chronic inflammation that is typical of aging and RA constitutes an additional handicap [1,3,23], which should be considered by health service providers when designing care plans for older persons.

The present study aimed to describe the prevalence of sarcopenia in RA patients aged ≥65 years and identify the risk factors associated with sarcopenia. This age group was chosen in order to better understand the risk factors of RA in older age. Consistent with findings from other studies, affected patients had a lower socioeconomic level and more comorbid conditions than controls from their setting [46]. Socioeconomic level may depend on occupational and residential exposure and lifestyle, and has traditionally been considered a major risk factor in RA [47] and in many comorbid conditions [48], including sarcopenia [49]. Comorbid conditions affected all organ systems and were associated with a high age-CCI, which is predictive of greater mortality at 10 years [30].

According to previous studies, sarcopenia affects 4.5–40% of patients with RA of all ages [5,11,12,13,20]. However, this variability is due to methodological differences, including the criteria applied for definition, cut-off points, and use of other technologies. In the present study, DXA was used to detect sarcopenia, which was defined following the criteria of EWGSOP2. These criteria are accepted internationally and are the most commonly used today [50], although they have not been widely applied in RA. In addition, as they are more specific, they reveal comparatively lower prevalence values in RA [51]. Moreover, DXA is considered the gold standard in clinical practice for non-invasive assessment of muscle mass, even though other techniques, such as magnetic resonance imaging, may be more precise (and more expensive). Given these conditions, the prevalence of sarcopenia in RA patients aged ≥65 years in the present study was 15.8%, compared with only 3.9% in healthy controls. Our results are consistent with those reported in other studies that use DXA and the EWGSOP2 criteria for detection and definition of sarcopenia [5,22,51], taking into account that our sample of patients was more exposed to sarcopenia owing to their age [19]. Notwithstanding, the prevalence detected in these patients did not prove to be much higher, probably owing to other factors, such as better control of inflammatory activity in our sample and less frequent use of glucocorticoids in favor of the more frequent prescription of bDMARDs. Consistent with most studies, bDMARDs improve appendicular muscle mass and function in almost half of patients with RA [52].

While all measures of body fat were elevated, it is worth highlighting the increase in the A/G ratio in RA patients. Even though this variable has received little attention in other studies on sarcopenia in RA, it warrants special attention because of its importance in affected patients. A high A/G ratio is a good indicator of central adiposity and is associated with a greater risk of vertebral fracture [53], cardiovascular disease, type 2 diabetes mellitus [54], and mortality than in the general population [55]. Furthermore, the A/G ratio is linked to the progression of sarcopenia and inflammation. This could be related to the data observed in our patients [56]. Nevertheless, these findings should be interpreted with caution, since gynoid and android fat measures that are taken using older versions of DXA, as was the case in our study, do not distinguish between subcutaneous fat (orthotopic) and the ectopic fat in the abdominal muscle fascia, and the mesenteric fat surrounding organs and blood vessels [57].

The loss of muscle mass with preservation of body weight is compensated by the increase in fatty tissue and is typical of rheumatoid cachexia, which is also associated with an increased risk of cardiovascular disease [58]. Specifically, sarcopenic obesity was recorded in 10% of the RA patients in the present study and was rare among the controls. These findings are consistent with those reported elsewhere [22], which highlight the major role of inflammation and fatty tissue in the pathogenesis of sarcopenia in RA: adipocytes undergo hypertrophy and hyperplasia and behave like an inflammatory tissue that produces excess levels of proinflammatory mediators, such as TNF-α, IL-12 and IL-6 [59,60], and leptin [16]. A chronically high concentration of proinflammatory cytokines over time promotes proteolysis, and together with low physical activity and poor nutritional status, increases the risk of sarcopenia and cardiovascular disease in affected patients [20,61,62].

In this context, it seems logical that the main independent risk factors identified in the present study are related to RA, nutrition, and physical activity. The factor most directly related to RA was disease duration, which was associated with an annual 10% incremental risk of sarcopenia. In this sense, several studies have found an association between sarcopenia and duration of arthritis [13,20]. Additionally, we found that the risk decreases proportionally in better nourished patients, as confirmed elsewhere [63]. These findings suggest that screening for sarcopenia and implementing interventions to improve nutritional status and physical function in RA patients may be important for preventing and managing sarcopenia and associated negative health outcomes.

Lastly, and no less important, we found that men had a higher risk of sarcopenia than women. While it is obvious that men have greater muscle mass than women, and that both begin to lose this mass after the age of 40 years, this accelerates in men after age 50, thus explaining why this difference is not observed in persons who are older [7,13]. The phenomenon can be explained by factors, such as a more significant reduction in growth hormone and testosterone level [64].

Although we did not observe an association between bDMARDs and sarcopenia, these drugs have a positive effect on grip strength and physical performance in most studies [52]. In addition to improving life expectancy [4], the high frequency of bDMARD prescription in our study (74%) led to a lower prevalence of sarcopenia.

Similarly, we did not find an association between sarcopenia and inflammatory values at the cut-off, such as DAS28-ESR and CRP, probably because the effect of inflammation on body composition grows over time, as opposed to occurring at a specific point in time. In order to evaluate this effect appropriately, it would be necessary to use a different design based on longitudinal data [65]. These findings are consistent with those reported elsewhere [13,19,22].

Our study is subject to a series of limitations, such as the relatively small sample size and the cross-sectional design. Some of the differences observed between cases and controls were not statistically significant—probably owing to the sample size—and should be interpreted with caution. Nevertheless, both cases and controls were well characterized, and good case definitions were used, thus enhancing internal validity and providing us with a more accurate picture of the associations between RA and sarcopenia. Additionally, as the study only included participants who were ≥65 years, the generalizability of our findings to younger populations is limited. On the other hand, there are other predictors of sarcopenia not included in our study, such as genomic predictors. Genomic predictors of sarcopenia based on previously discovered genome-wide significant SNPs, associated with handgrip strength, appendicular lean mass, and walking pace, have been described [66]. Finally, the study participants were selected from four teaching centers in Spain that are relatively close to one another, thus potentially restricting the generalizability of our findings to areas outside Spain.

## 5. Conclusions

Our results suggest that RA patients aged ≥65 years may be at increased risk for sarcopenia and malnutrition, especially in the case of men with long-standing disease and worse nutritional values. These findings could have important implications for the management and care of older patients with RA, although further research is needed to confirm and expand our data.

## Figures and Tables

**Table 1:** Baseline epidemiological characteristics and comorbidities of older RA patients and controls.

	RA Patients N = 76	Controls N = 76	*p*-Value
Age, yrs, mean (SD)	71.0 (4.8)	71.2 (4.9)	0.726
65–69 yrs	32 (42.1)	29 (38.2)	
70–79 yrs	40 (52.6)	42 (55.3)	
80–90 yrs	4 (5.3)	5 (6.6)	
Women, n (%)	60 (78.9)	60 (78.9)	1.000
Smoking status			0.121
Non-smokers, n (%)	54 (71)	57 (75)	
Smokers, n (%)	6 (7.9)	11 (14.5)	
Former smokers, n (%)	16 (21.1)	8 (10.5)	
Alcohol intake, n (%)	12 (15.8)	17 (22.5)	0.195
Educational level			<0.001
No studies	9 (11.8)	1 (1.3)	
Primary studies	55 (72.4)	41 (53.9)	
Secondary studies	10 (13.2)	24 (31.6)	
Higher education	2 (2.6)	10 (13.2)	
Economic level			0.003
No income, n (%)	12 (15.8)	5 (6.6)	
Income < EUR 1500, n (%)	51 (67.1)	40 (52.6)	
Income ≥ EUR 1500, n (%)	13 (17.1)	31 (40.8)	
Comorbidities			
High blood pressure, n (%)	43 (56.6)	21 (27.6)	<0.001
Diabetes mellitus, n (%)	13 (17.1)	8 (10.5)	0.240
Dyslipidemia, n (%)	30 (39.5)	19 (25.0)	0.056
Cardiovascular disease, n (%)	4 (5.3)	1 (1.3)	0.172
Osteoporosis, n (%)	15 (19.7)	4 (5.3)	0.007
Asthma, n (%)	1 (7.9)	0 (0.0)	0.012
Other comorbidities, n (%)	44 (57.9)	29 (38.2)	0.015
Age-CCI, median (p25–p75)	3.0 (3.0–4.0)	3.0 (2.0–3.0)	<0.001
Estimated 10-year survival (%), median (p25–p75)	77.0 (53.0–77.0)	77.0 (77.0–90.0)	<0.001

Abbreviations: RA, rheumatoid arthritis; age-CCI, age-adjusted Charlson Comorbidity Index.

**Table 2:** Baseline clinical characteristics and treatments in RA patients and controls.

	RA Patients N = 76	Controls N = 76	*p*-Value
Duration of RA, years, mean (SD)	18 (7.8)	-	
RF positive > 10 IU/mL, n (%)	57 (75.0)	0	
ACPA positive > 20 IU/mL, n (%)	55 (72.4)	0	
CRP, mg/L, median (p25–p75)	3.3 (2.1–6.0)	2.7 (1.1–4.0)	0.002
DAS28-ESR at cut-off, score 0–10, mean (SD)	2.9 (1.1)	-	
Remission or low activity, n (%)	49 (64.5)		
Moderate or high activity, n (%)	27 (35.5)		
HAQ-DI, score 0–3, mean (SD)	1.282 (0.798)	-	
Treatments			
NSAIDs, n (%)	32 (42.1)	6 (7.9)	<0.001
Analgesics, n (%)	1.0 (0.7)	0.2 (0.5)	<0.001
csDMARDs, n (%)	45 (59.2)	0 (0.0)	<0.001
No. of csDMARDs, median (p25–p75)	1.0 (0–1.7)	0	<0.001
bDMARDs, n (%)	56 (73.7)	0 (0.0)	<0.001
No. of bDMARDs, median (p25–p75)	4.0 (0.0–7.0)	0	<0.001
Glucocorticoids at cut-off, n (%)	44 (57.9)	0	<0.001
Polypharmacy, n (%)	69 (90.8)	23 (30.3)	<0.001
No. of drugs, median (p25–p75)	8.0 (6.0–11.0)	3.0 (2.0–4.7)	<0.001

Abbreviations: RA, rheumatoid arthritis; RF, rheumatoid factor; ACPA, anti–citrullinated protein antibodies; CRP, C-reactive protein; DAS28-ESR, 28-joint Disease Activity Score with erythrocyte sedimentation rate; HAQ-DI, Health Assessment Questionnaire Disability Index; NSAIDs, non-steroidal anti-inflammatory drugs; DMARDs, disease-modifying antirheumatic drugs; csDMARDs, conventional synthetic disease-modifying antirheumatic drugs; bDMARDs, biologic disease-modifying antirheumatic drugs.

**Table 3:** Anthropometric data, nutritional status, strength, and performance of RA patients and controls.

	RA Patients N = 76	Controls N = 76	*p*-Value
Anthropometric measurements			
BMI, kg/m^2^, mean (SD)	28.1 (5.0)	28.1 (4.5)	0.296
Underweight	1 (1.3)	1 (1.3)	
Normal weight	14 (18.4)	16 (21.1)	
Overweight	31 (40.8)	38 (50.0)	
Obesity, n (%)	30 (39.5)	21 (27.6)	0.252
Class I obesity	21 (27.6)	12 (15.8)	
Class II obesity	6 (7.9)	9 (11.8)	
Class II obesity	3 (3.9)	0	
Right arm circumference, cm	29.5 (4.5)	29.9 (4.5)	0.422
Left arm circumference, cm	29.6 (4.5)	29.9 (4.5)	0.714
Left triceps skinfold, mm	14.0 (10.3–19.0)	17.2 (11.8–23.0)	0.034
Right triceps skinfold, mm	14.0 (10.2–17.9)	15.5 (11.8–23.5)	0.049
Nutrition			
MNA, mean (SD)	12.3 (2.0)	12.9 (1.6)	0.057
Malnutrition, n (%)	24 (31.6)	17 (22.4)	0.201
Total proteins, g/L, mean (SD)	6.8 (0.5)	6.9 (0.7)	0.081
Albumin, g/L, mean (SD)	4.1 (0.4)	4.3 (0.7)	0.139
Hemoglobin, mg/dL, median (p25–p75)	13.2 (12.2–14.1)	13.9 (12.9–14.7)	0.322
Calcium, mg/dL, mean (SD)	11.8 (13.8)	9.5 (0.6)	0.182
Vitamin B12, pg/mL, median (p25–p75)	315.5 (258.2–404.2)	397.0 (305.0–501.0)	0.006
Vitamin D, ng/mL, mean (SD)	30.8 (15.3)	28.8 (14.3)	0.456
Strength and performance			
Functional class			<0.001
Steinbrocker I, n (%)	22 (28.9)	67 (88.2)	
Steinbrocker II, n (%)	38 (50.0)	8 (10.5)	
Steinbrocker III, n (%)	15 (19.7)	1 (1.3)	
Steinbrocker IV, n (%)	1 (1.3)	0 (0.0)	
Physical frailty, n (%)	31 (40.1)	12 (15.8)	<0.001
EuroQol—VAS, median (p25–p75)	55.0 (41.2–69.0)	75.0 (60.0–80.0)	<0.001
EuroQol 5D-5L, median (p25–p75) (0–1)	0.53 (0.31–0.71)	1.0 (0.78–1.0)	0.001
IPAQ, METs, median (p25–p75)	260.0 (0.0–630.0)	594.0 (0.0–1173.7)	0.002
Handgrip strength, kg, median (p25–p75)	16.8 (10.4–21.9)	25.0 (20.0–35.5)	<0.001
Reduced handgrip strength, n (%)	65 (85.5)	42 (55.3)	<0.001
Gait speed, m/s, median (p25–p75)	1.2 (1.0–1.5)	0.9 (0.8–1.2)	<0.001
Low speed, n (%)	9 (11.8)	16 (21.1)	0.126
SPPB, median (p25–p75)	7.0 (5.5–9.0)	10.0 (9.0–11.0)	<0.001
SARC-F questionnaire (0–10), median (p25–p75)	5.0 (3–6)	1.0 (0–2)	<0.001
High SARC-F, n (%)	53 (69.7)	12 (15.8)	<0.001
Body composition			
Appendicular LM, kg, median (p25–p75)	14.7 (13.8–16.9)	15.5 (15.4–18.9)	0.210
Appendicular FFMI, kg/m^2^, median (p25–p75)	6.0 (5.3–7.0)	6.2 (5.6–7.2)	0.074
Appendicular FM, kg, median (p25–p75)	12.8 (11.7–14.1)	11.09 (10.06–13.6)	0.104
FMI, kg/m^2^, mean (SD)	5.2 (1.8)	4.7 (1.7)	0.104
High FMI *, n (%)	43 (56.6)	23 (41.8)	0.095
Sarcopenia EWGSOP2, n (%)			
Probable sarcopenia, n (%)	35 (46.1)	11 (14.5)	<0.001
Confirmed sarcopenia, n (%)	12 (15.8)	3 (3.9)	0.014
Severe sarcopenia	1 (1.3)	0	1.000
Sarcopenic obesity †, n (%)	8 (10.5)	1 (1.3)	0.016
Osteosarcopenia ‡, n (%)	3 (4.1)	0	0.076

Abbreviations: RA, rheumatoid arthritis; BMI, body mass index; MNA, Mini Nutritional Assessment; EuroQol-5D-5L, European Quality of Life 5-Dimension 5-Level; VAS, visual analog scale; IPAQ, International Physical Activity Questionnaire; SPPB, Short Physical Performance Battery; SARC-F, Strength, Assistance in walking, Rise from a chair, Climb stairs, and Falls; LM, lean mass; FFMI, fat-free mass index; FM, fat mass; FMI, fat mass index; EWGSOP2; Revised European Working Group on Sarcopenia in Older People criteria. * FMI ≥ 13 for women and ≥9 for men; † Confirmed sarcopenia plus high FMI. ‡ Sarcopenia plus osteoporosis in the hip or spine.

**Table 4:** Univariate and multivariate logistic regression analysis. Dependent variable: confirmed sarcopenia in patients with RA (R2 Nagelkerke = 0.722).

		Univariate			Multivariate	
Predictor	OR	95% CI	*p*-Value	OR	95% CI	*p*-Value
Male sex	0.3	0.1–1.0	0.057	9.3	1.1–80.4	0.042
Age, yrs	0.9	0.97–0.9	<0.001			
RA duration, yrs	0.9	0.9–0.9	<0.001	1.1	1.0–1.2	0.012
MNA screening	0.9	0.8–0.9	<0.001	0.7	0.5–0.9	0.042
SPPB	0.8	0.7–0.9	<0.001			
DAS28-ESR	0.6	0.5–0.8	<0.001			

Variables specified in step 1: Sex, Age, RA duration, DAS28-ESR, MNA screening, SPPB. Abbreviations: OR = odd ratio; MNA = Mini Nutritional Assessment; SPPB = Short Physical Performance Battery; DAS-ESR, 28-joint Disease Activity Score with erythrocyte sedimentation rate.

## Data Availability

The datasets used and/or analysed in the present study are available from the corresponding author upon reasonable request.

## Supplementary Materials

The following supporting information can be downloaded at: https://www.mdpi.com/article/10.3390/nu15112440/s1, Figure S1: Logistic regression analysis plot. Dependent variable: Sarcopenia obesity in patients with RA. Abbreviation: MNA = Mini Nutritional Assessment; Table S1: Body composition in patients and controls; Table S2: Characteristics of sarcopenic RA patients compared with non-sarcopenic RA patients; Table S3: Characteristics of sarcopenic obesity RA patients compared with non-sarcopenic obesity RA patients; Table S4: Body composition in sarcopenic and non-sarcopenic RA patients. Table S5. Body composition in sarcopenic obesity and non-sarcopenic obesity RA patients; Table S6: Univariate and multivariate logistic regression analysis. Dependent variable: Sarcopenic obesity in patients with RA (R2 Nagelkerke = 0.736).
