# Supplementary material for: Sarcopenia and Nutrition in Elderly Rheumatoid Arthritis Patients: A Cross-Sectional Study to Determine Prevalence and Risk Factors
Source: Nutrients. 2023 May 24;15(11):2440. doi: 10.3390/nu15112440 (PMC10255254; doi:10.3390/nu15112440)
Supplement: Supplementary file 1 [file nutrients-15-02440-s001.zip › nutrients-2416132-supplementary.pdf]

Supplementary Materials:

Supplementary Table S1: Body composition in patients and controls.

**Body Composition.**		**RA Patients N = 76**	**Controls N = 76**	***p*-Value**
Total body	Tissue mass, kg, median (p25–p75)	72.5 (64.2–81.2)	67.5 (61.1–76.6)	0.124
	FM, %, mean (SD) (adiposity)	44.5 (7.7)	41.0 (8.1)	0.946
	FM, kg, mean (SD)	32.6 (8.4)	30.0 (9.1)	0.610
	LM, kg, median (p25–p75)	40.4 (8.4)	42.3 (7.8)	0.467
	BMC, g, median (p25–p75)	2340.1 (2016.0–3341.7)	2937.0 (2190.0–3957.0)	0.006
	FMI, kg/m^2, mean (SD)	13.0 (3.9)	11.5 (4.2)	0.282
	FFMI, kg/m^2, median (p25–p75)	15.3 (15.1–16.5)	15.6 (15.1–16.7)	0.696
	FMR, mean (SD)	0.9 (0.2)	0.8 (0.2)	0.017
Appendicular	Tissue mass, kg, median (p25–p75)	29.1 (28.5–31.6)	28.4 (28.0–32.8)	0.624
	FM, %, mean (SD)	42.7 (9.6)	39.7 (9.8)	0.103
	FM, kg, median (p25–p75)	12.8 (11.7–14.1)	11.09 (10.06–13.6)	0.104
	LM, kg, median (p25–p75)	14.7 (13.8–16.9)	15.5 (15.4–18.9)	0.210
	BMC, g, median (p25–p75)	1023.5 (1038.4–1195.8)	1152 (1108.5–1322.7)	0.629
	FMI, kg/m2, mean (SD)	5.2 (1.8)	4.7 (1.7)	0.104
	FFMI, kg/m2, median (p25–p75)	6.0 (5.3–7.0)	6.2 (5.6–7.2)	0.074
Trunk	Tissue mass, kg, median (p25–p75)	37.4 (35.4–39.4)	35.4 (34.3–40.3)	0.450
	FM, %, mean (SD)	47.6 (7.2)	45.6 (7.4)	0.387
	FM, kg, mean (SD)	18.0 (5.0)	17.2 (5.3)	0.355
	LM, kg, median (p25–p75)	19.5 (18.7–20.8)	19.4 (19.0–21.7)	0.755
	BMC, g, median (p25–p75)	676 (651.8–751.3)	657.5 (657.4–838.5)	0.315
	FMI, kg/m2, mean (SD)	2.8 (1.0)	2.6 (1.1)	0.256
	FFMI, kg/m2, mean (SD)	7.7 (1.2)	7.7 (1.4)	0.656
	Android fat mass (%), mean (SD)	51.5 (7.5)	50.0 (7.5)	0.131
	Gynoid fat mass (%), mean (SD)	49.0 (9.2)	55.9 (16.0)	0.005
	A/G ratio, median (p25–p75)	1.0 (0.9–1.2)	0.9 (0.8–1.1)	<0.001

Abbreviations: RA, rheumatoid arthritis; BMC, bone mass content; LM, lean mass; FFMI, fat-free mass index; FM, fat mass; FMI, fat mass index; FMR, fat-to-muscle ratio; A/G android/gynoid.

Supplementary Table S2. Characteristics of sarcopenic RA patients compared with non-sarcopenic RA patients.

	Sarcopenic RA Patients (N = 12)	Non-Sarcopenic RA Patients (N = 64)	*p*-Value
Age, yrs, median (p25–p75)	72.5 (69.2–77.0)	70.0 (67.0–63.0)	0.094
Women, n (%)	8 (66.7)	52 (81.3)	0.255
Smoking status			0.929
Non-smokers, n (%)	8 (66.7)	46 (71.9)	
Smokers, n (%)	1 (8.3)	5 (7.8)	
Former smokers, n (%)	3 (25.0)	13 (20.3)	
Alcohol intake, n (%)	3 (25.0)	9 (14.1)	0.390
Educational level			0.880
No studies	1 (8.3)	8 (12.5)	
Primary studies	9 (75.0)	46 (71.9)	
Secondary studies	2 (16.7)	8 (12.5)	
Higher education	0	2 (3.1)	
Economic level			0.600
No income, n (%)	1 (8.3)	11 (17.2)	
Income <EUR 1500, n (%)	8 (66.7)	43 (67.2)	
Income >EUR 1500, n (%)	3 (25.0)	10 (15.6)	
Comorbidities			
High blood pressure, n (%)	5 (25.7)	38 (61.3)	0.081
Diabetes mellitus, n (%)	2 (16.7)	11 (17.2)	1.000
Dyslipidemia, n (%)	5 (41.7)	25 (39.1)	1.000
Cardiovascular disease, n (%)	0	4 (6.3)	1.000
Osteoporosis, n (%)	3 (25.0)	12 (18.8)	0.695
Asthma, n (%)	1 (8.3)	5 (7.8)	1.0
Other comorbidities, n (%)	6 (50.0)	38 (59.4)	0.546
Age-CCI, median (p25–p75)	3.0 (2.2–4.0)	3.0 (3.0–4.0)	0.872
Estimated 10-year survival (%), median (p25–p75)	77.0 (53.0–77.0)	77.0 (77.0–90.0)	0.872
RA variables			
Duration of RA, years, median (p25–p75)	22.5 (15.2–33.2)	15.0 (12.0–21.7)	0.018
RF positive >10 IU/mL, n (%)	11 (91.7)	54 (84.4)	0.510
ACPA positive >20 IU/mL, n (%)	10 (83.3)	47 (73.4)	0.719
CRP, mg/L, mean (SD)	10 (83.3)	45 (70.3)	0.492
DAS28-ESR at cut-off, score 0–10, median (p25–p75)	3.2 (2.6–4.1)	2.6 (1.9–3.3)	0.054
Remission or low activity, n (%)	7 (58.3)	20 (31.3)	0.072
Moderate or high activity, n (%)	5 (41.7)	44 (68.8)	0.072
HAQ-DI, score 0–3, mean (SD)	1.4 (0.8)	1.3 (0.8)	0.727
Treatments			
NSAIDs, n (%)	5 (41.7)	27 (42.2)	0.973
Analgesics, median (p25–p75)	1.0 (1.0–0.5)	1.0 (1.0–2.0)	0.597
Conventional synthetic DMARDs, n (%)	7 (58.3)	38 (59.4)	1.0
Biological DMARDs, n (%)	8 (66.7)	48 (75.0)	0.722
Glucocorticoids at cut-off, n (%)	8 (66.7)	36 (56.3)	0.502
Polypharmacy, n (%)	13 (92.9)	56 (90.3)	0.767
No. of drugs, median (p25–p75)	8.0 (6.2–11.7)	8.0 (6.0–10.7)	0.567
Anthropometric measurements			
BMI, kg/m^2, mean (SD)	26.5 (4.2)	29.4 (5.0)	0.063
Obesity, n (%)	5 (35.7)	25 (40.3)	0.166
Right arm circumference, cm	28 (24.5–29.7)	29 (27.5–31.0)	0.063

Left arm circumference, cm	27.2 (24.2–30.0)	29 (27.0–31.0)	0.132
Left triceps skinfold, mm	11.4 (9.2–11.5)	14.5 (11.6–19.1)	0.095
Right triceps skinfold, mm	11.5 (9.2–14.0)	15 (11.2–19.4)	0.057
Nutrition	11.0 (10.0–13.0)	13.0 (11.0–14.0)	0.029
MNA, mean (SD)	7 (58.3)	17 (26.6)	0.030
Malnutrition, n (%)	6.9 (6.6–7.2)	6.8 (6.-7.0)	0.321
Total proteins, g/L, mean (SD)	4.2 (0.5)	4.1 (0.4)	0.813
Albumin, g/L, median (p25–p75)	4.3 (3.5–4.7)	4.1 (3.9–4.5)	0.820
Hemoglobin, mg/dL, median (p25–p75)	13.2 (12.1–13.9)	13.1 (12.2–14.1)	0.194
Calcium, mg/dL, median (p25–p75)	8.9 (8.8–9.8)	9.4 (9.1–9.9)	0.866
Vitamin B12, pg/mL, median (p25–p75)	311 (272.5–414.5)	320 (258.0–405.0)	0.550
Vitamin D, ng/mL, median (p25–p75)	21.5 (17.5–32.4)	28.4 (18.4–42.9)	0.029
Strength and performance			
Functional class			0.127
Steinbrocker I, n (%)	44 (33.3)	18 (28.1)	
Steinbrocker II, n (%)	5 (41.7)	33 (51.6)	
Steinbrocker III, n (%)	2 (16.7)	13 (20.3)	
Steinbrocker IV, n (%)	1 (8.3)	0	
Older people with frailty, n (%)	9 (100)	39 (81.3)	0.328
EuroQol—VAS, median (p25–p75)	57.5 (32.5–70.0)	55.0 (45.0–6.7)	0.807
EuroQol 5D-5L, median (p25–p75) (0–1)	0.4 (0.1–0.7)	0.5 (0.3–0.7)	0.154
IPAQ, METs, median (p25–p75)	172.5 (0.0–399.0)	270.0 (0.0–676.5)	0.002
Handgrip strength, kg, median (p25–p75)	9.8 (8.75–13.5)	17.55 (14.175–23.125)	0.021
Reduced handgrip strength, n (%)	14 (100)	51 (82.3)	0.088
Gait speed, m/s, median (p25–p75)	1.52 (0.675–1.69)	1.2 (1–1.532)	0.135
Low speed, n (%)	9.8 (8.75–13.5)	17.55 (14.175–23.125)	0.021
SPPB, median (p25–p75)	6.0 (4.5–7.0)	8.0 (6.0–9.0)	0.088
SARC-F questionnaire (0–10), median (p25–p75)	5.2 (4.0–6.7)	5.0 (2.2–6.0)	0.135

Abbreviations: RA, rheumatoid arthritis; BMI, body mass index; MNA, Mini Nutritional Assessment; EuroQol 5D-5L, European Quality of Life 5-Dimension 5-Level; VAS, visual analog scale; IPAQ, International Physical Activity Questionnaire; SPPB, Short Physical Performance Battery; SARC-F, Strength, Assistance in walking, Rise from a chair, Climb stairs, and Falls.

Supplementary Table S3. Characteristics of sarcopenic obesity RA patients compared with non-sarcopenic obesity RA patients.

	**Sarcopenic Obesity RA Patients (N = 8)**	**Non-Sarcopenic Obesity RA Patients (N = 68)**	***p*-Value**
Age, yrs, median (p25–p75)	72.5 (67.7–77.0)	70.5 (67.0–73.0)	0.262
Women, n (%)	5 (62.5)	55 (80.9)	0.228
Smoking status			0.673
Non-smokers, n (%)	6 (75.0)	48 (70.6)	
Smokers, n (%)	0 (0.0)	6 (8.8)	
Former smokers, n (%)	2 (25.0)	14 (20.6)	
Alcohol intake, n (%)	1 (12.5)	11 (16.2)	0.787
Educational level			0.726
No studies	1 (12.)	8 (11.8)	
Primary studies	5 (62.5)	50 (73.5)	
Secondary studies	2 (25.0)	8 (11.8)	
Higher education	0	2 (2.9)	
Economic level			0.402
No income, n (%)	0 (0.0)	12 (17.6)	

Income <EUR 1500, n (%)	6 (75.0)	45 (66.2)	
Income >EUR 1500, n (%)	2 (25.0)	11 (16.2)	
Comorbidities			
High blood pressure, n (%)	5 (62.5)	38 (55.9)	0.721
Diabetes mellitus, n (%)	1 (12.5)	12 (17.6)	0.715
Dyslipidemia, n (%)	4 (50.0)	26 (38.2)	0.520
Cardiovascular disease, n (%)	0(0.0)	4 (5.9)	0.481
Osteoporosis, n (%)	2 (25.0)	13 (19.1)	0.693
Asthma, n (%)	1 (12.5)	5 (7.4)	0.610
Other comorbidities, n (%)	4 (50.0)	40 (58.8)	0.633
Age-CCI, median (p25–p75)	3.0 (3.0–4.750)	3.0 (3.0–4.0)	0.519
Estimated 10-year survival (%), median (p25–p75)	77.0 (2.0–77.0)	77.0 (53.0–77.0)	0.519
RA variables			
Duration of RA, years, median (p25–p75)	21.0 (14.-35.5)	15.5 (12.0–22.0)	0.081
RF positive >10 IU/mL, n (%)	6 (75.0)	51 (75.0)	1.000
ACPA positive >20 IU/mL, n (%)	6 (75.0)	49 (72.1)	0.860
CRP, mg/L, median (p25–p75)	3.2 (1.5–5.0)	3.7 (2.2–6.0)	0.509
DAS28-ESR at cut-off, score 0–10, mean (SD)	3.2 (0.7)	2.8 (1.1)	0.377
Remission or low activity, n (%)	4 (50)	45 (66.2)	0.366
Moderate or high activity, n (%)	4 (50)	23 (33.8)	0.366
HAQ-DI, score 0–3, mean (SD)	1.3 (0.8)	1.2 (0.8)	0.872
Treatments			
NSAIDs, n (%)	3 (37.5)	29 (42.6)	0.780
Analgesics, median (p25–p75)	1.0 (1.0–1.0)	1.0 (1.0–2.0)	0.227
Conventional synthetic DMARDs, n (%)	3 (37.5)	42 (61.8)	0.187
Biological DMARDs, n (%)	5 (62.5)	51 (75.0)	0.448
Glucocorticoids at cut-off, n (%)	5 (62.5)	39 (57.4)	0.780
Polypharmacy, n (%)	7 (87.5)	62 (91.2)	0.734
No. of drugs, median (p25–p75)	8.0 (5.2–11.0)	8.0 (6.0- 11.0)	0.652
Anthropometric measurements			
BMI, kg/m^2, mean (SD)	27.1 (3.7)	29.1 (5.0)	0.299
Obesity, n (%)	2 (25.0)	28 (41.2)	0.376
Right arm circumference, cm median (p25–p75)	28 (26.1–28.7)	29.o (27.5–31.0)	0.095
Left arm circumference, cm median (p25–p75)	27.2 (25.3–29.6)	29.0 (27.0–31.0)	0.194
Left triceps skinfold, mm median (p25–p75)	11.5 (9.4–14.7)	15.0 (10.2–18.7)	0.226
Right triceps skinfold, mm median (p25–p75)	11.7 (9.5–18.5)	14.0 (10.-18.9)	0.343
Nutrition			
MNA, mean (SD)	11.6 (1.5)	12.3 (2.0)	0.304
Malnutrition, n (%)	5 (62.5)	19 (27.9)	0.047
Total proteins, g/L, mean (SD)	6.8 (0.3)	6.7 (0.4)	0.603
Albumin, g/L, median (p25–p75)	4.3 (4.3–4.7)	4.1 (3.8–4.5)	0.195
Hemoglobin, mg/dL, median (p25–p75)	13.2 (12.3–14.1)	13.1 (12.2–14.1)	0.748
Calcium, mg/dL, median (p25–p75)	8.9 (8.6–9.6)	9.4 (9.0–9.9)	0.139
Vitamin B12, pg/mL, median (p25–p75)	363.0 (299.0–400.0)	315.5 (257.0–402.7)	0.574
Vitamin D, ng/mL, median (p25–p75)	21.7 (19.8–47.5)	28.2 (17.5–41.6)	0.988
Strength and performance			
Functional class			0.026
Steinbrocker I, n (%)	3 (37.5)	19 (27.9)	
Steinbrocker II, n (%)	3 (37.5)	35 (51.5)	
Steinbrocker III, n (%)	1 (12.5)	14 (20.6)	
Steinbrocker IV, n (%)	1 (12.5)	0.0	
Older people with frailty, n (%)	6 (100)	42 (82.4)	0.262

EuroQol—VAS, median (p25–p75)	57.5 (25.0–70.0)	55.0 (45.0–65.7)	0.892
EuroQol 5D-5L, median (p25–p75) (0–1)	0.4 (0.3–0.7)	0.5 (0.2–0.7)	0.648
IPAQ, METs, median (p25–p75)	206.0 (0.0–399.0)	260.0 (0.0–676.5)	0.509
Handgrip strength, kg, median (p25–p75)	10.1 (8.-17.3)	17.0 (12.-22.0)	0.054
Reduced handgrip strength, n (%)	8 (100)	27 (39.7)	0.001
Gait speed, m/s, median (p25–p75)	1. (1.-1.6)	1.2 (0.9–1.)	0.347
Low speed, n (%)	1 (12.5)	8 (11.8)	0.951
SPPB, median (p25–p75)	6.5 (4.7–7.5)	7.0 (6.0–9-0)	0.254
SARC-F questionnaire (0–10), median (p25–p75)	5. (4.2–6.0)	5.0 (3.0–6.0)	0.719

Abbreviations: RA, rheumatoid arthritis; BMI, body mass index; MNA, Mini Nutritional Assessment; EuroQol 5D-5L, European Quality of Life 5-Dimension 5-Level; VAS, visual analog scale; IPAQ, International Physical Activity Questionnaire; SPPB, Short Physical Performance Battery; SARC-F, Strength, Assistance in walking, Rise from a chair, Climb stairs, and Falls.

Supplementary Table S4. Body composition in sarcopenic and non-sarcopenic RA patients.

Body composition		Sarcopenic RA Patients (N = 12)	Non-Sarcopenic RA Patients (N = 64)	*p*-Value
Total body	Tissue mass, kg, median (p25–p75)	65.3 (52.5–74.5)	73.4 (65.3–83.7)	0.019
	FM, %, mean (SD) (adiposity)	44.2 (5.7)	44.0 (7.2)	0.683
	FM, kg, mean (SD)	28.8 (8.2)	33.3 (7.7)	0.073
	LM, kg, median (p25–p75)	33.8 (32–37.2)	38.7 (33.4–48)	0.052
	BMC, g, median (p25–p75)	2043.1 (1842.5–3222.2)	2376.9 (2119.5–3341.7)	0.122
	FMI, kg/m2, mean (SD)	11.8 (3)	13.4 (3.7)	0.161
	FFMI, kg/m2, median (p25–p75)	5.1 (4.8–5.7)	6.3 (5.5–6.3)	0.038
	FMR, mean (SD)	0.8 (0.1)	0.8 (0.2)	0.544
Appendicular	Tissue mass, kg, median (p25–p75)	26.4 (21.3–30.1)	29.3 (26.3–34.7)	0.010
	FM, %, mean (SD)	44 (5.7)	43.1 (9.1)	0.748
	FM, kg, median (p25–p75)	10.5 (8–13.8)	13.1 (9.9–13.1)	0.066
	LM, kg, median (p25–p75)	12.6 (11.2–13.9)	14.8(13.1–18)	<0.001
	BMC, g, median (p25–p75)	934 (858–1029.5)	1024 (907.2–1250.7)	0.073
	FMI, kg/m2, mean (SD)	4.5 (1.1)	5.4 (1.9)	0.128
	FFMI, kg/m2, median (p25–p75)	5.1 (4.8–5.8)	6.3 (5.5–7.2)	0.001
Trunk	Tissue mass, kg, median (p25–p75)	30.5 (26.7–35.9)	37.5 (31.7–42.7)	0.004
	FM, %, mean (SD)	46.9 (6.9)	48.5 (6.8)	0.344
	FM, kg, mean (SD)	14.9 (4.3)	18.5 (4.7)	0.018
	LM, kg, median (p25–p75)	16.8 (16.1–19.1)	19.9 (17–23.4)	0.022
	BMC, g, median (p25–p75)	547 (461.5–758.5)	626 (490–742.2)	0.326
	FMI, kg/m2, mean (SD)	2.5 (0.8)	3.0 (1.0)	0.153
	FFMI, kg/m2, mean (SD)	7.2 (0.7)	8.0 (1.2)	0.033
	Android fat mass (%), mean (SD)	49.4 (6.8)	51.9 (7.6)	0.302
	Gynoid fat mass (%), mean (SD)	49.3 (7.3)	48.9 (9.5)	0.877
	A/G ratio, median (p25–p75)	0.9 (0.9–1.0)	1.0 (0.9–1.1)	0.205

Abbreviations: RA, rheumatoid arthritis; BMC, bone mass content; LM, lean mass; FFMI, fat-free mass index; FM, fat mass; FMI, fat mass index; FMR, fat-to-muscle ratio; A/G, android/gynoid.

Supplementary Table S5. Body composition in sarcopenic obesity and non-sarcopenic obesity RA patients.

Body Composition	Sarcopenic Obesity RA Patients (N = 8)	Non-Sarcopenic Obesity RA Patients (N = 68)	*p*-Value

Total body	Tissue mass, kg, median (p25–p75)	71.8 (57.8–79.1)	72.5 (64.7–82.3)	0.446
	FM, %, mean (SD) (adiposity)	46.1 (4.4)	44.6 (7.3)	0.774
	FM, kg, mean (SD)	32.2 (6.6)	32.6 (8)	0.600
	LM, kg, median (p25–p75)	36.7 (32.7–41.6)	37.8 (33.4–46.7)	0.472
	BMC, g, median (p25–p75)	2246.7 (1958.6–3222.2)	2340.7 (2047.5–3341.7)	0.537
	FMI, kg/m2, mean (SD)	13.2 (2.1)	13.1 (3.7)	0.565
	FFMI, kg/m2, median (p25–p75)	14.9 (14.1–16.7)	15.4 (13.9–18.0)	0.554
	FMR, mean (SD)	0.9 (0.1)	0.8 (0.2)	
Appendicular	Tissue mass, kg, median (p25–p75)	28.1 (23.5–30.6)	29.2 (25.9–34.7)	0.168
	FM, %, mean (SD)	42.8 (5.9)	42.9 (8.9)	0.748
	FM, kg, median (p25–p75)	12.4 (8.7–12.4)	13 (9.8–15.2)	0.407
	LM, kg, median (p25–p75)	12.6 (11.3–14.1)	14.7 (13.17.7)	0.016
	BMC, g, median (p25–p75)	945 (923–1079.2)	1014.3 (898.1–1213.3)	0.361
	FMI, kg/m2, mean (SD)	4.8 (0.9)	5.3 (1.9)	0.456
	FFMI, kg/m2, median (p25–p75)	5.1 (4.8–5.8)	6.2 (5.4–7.1)	0.021
Trunk	Tissue mass, kg, median (p25–p75)	31.4 (29.9–40.1)	37.4 (30.8–41.7)	0.192
	FM, %, mean (SD)	49.8 (4.7)	48.0 (7.0)	0.774
	FM, kg, mean (SD)	16.9 (3.5)	18.1 (4.9)	0.600
	LM, kg, median (p25–p75)	18.1 (16.5–20.1)	19.5 (16.7–22.8)	0.310
	BMC, g, median (p25–p75)	607 (546.5–892)	604 (478.2–731)	0.642
	FMI, kg/m2, mean (SD)	2.9 (0.7)	2.0 (1.0)	0.839
	FFMI, kg/m2, mean (SD)	7.5 (0.7)	7.9 (1.2)	0.379
	Android fat mass (%), mean (SD)	52.3 (4.0)	51.4 (7.8)	0.761
	Gynoid fat mass (%), mean (SD)	50.3 (7.8)	48.8 (9.3)	0.813
	A/G ratio, median (p25–p75)	1.0 (0.9–1.1)	1.0 (0.9–1.1)	0.960

Abbreviations: RA, rheumatoid arthritis; BMC, bone mass content; LM, lean mass; FFMI, fat-free mass index; FM, fat mass; FMI, fat mass index; FMR, fat-to-muscle ratio; A/G, android/gynoid.

Supplementary Table S6. Univariate and multivariate logistic regression analysis. Dependent variable: Sarcopenic obesity in patients with RA (R2 Nagelkerke = 0.736).

	**Univariate**			**Multivariate**		
Predictor	OR	95% CI	*p*-value	OR	95% CI	*p*-value
Male sex	0.2	0.0–0.8	0.022	4.3	0.7–25.1	0.103
Age, yrs	0.9	0.97–0.9	<0.001	0.9	0.8–0.9	0.006
RA duration (Sqrt yrs)	0.6	0.5–0.7	<0.001	1.1	1.0–1.2	0.028
Manutrition	0.3	0.1–0.7	0.008	4.4	0.9–22.4	0.072

Variables specified in step 1: Sex, Age, RA duration, Malnutrition. Abbreviations: OR = odd ratio. Sqrt yrs = square root of years.

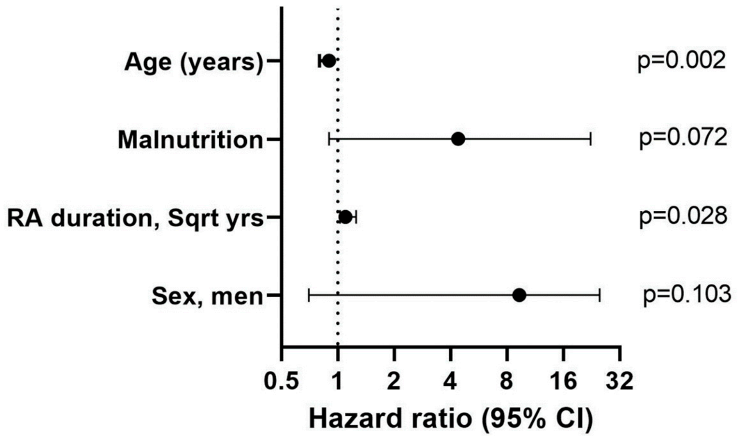

Supplementary Figure S1. Logistic regression analysis plot. Dependent variable: Sarcopenia obesity in patients with RA. Abbreviation: MNA = Mini Nutritional Assessment.
